# 

**Published:** 1897-07

**Authors:** 


					﻿CONTENTS.
ORIGINAL ARTICLES:
Ovarian Abscess; Operation,Recovery.................... 317
Laryngeal Diphtheria, Treated by Antitoxin and Calomel Fumi-
gation............................................... 321
SELECTIONS AND ABSTRACTS:
Abscesses of Both Kidneys Due to Chronic Cystitis, Abscesses
Opened and Drainage kept up, Steady Improvement...... 325
The use of Subcutaneous Injections of Artificial Serum in
Eclamptic Albuminura................................. 327
A Pathognomonic Sign of Acute Miliary Tuberculosis of the
Lungs................................................ 329
Death by Lightning...................................   330
What can the Country Doctor do to Prevent Typhoid Fever.	339
Water Treatment for Atrophy............................ 340
The Indigestion of Breast Babies ...................... 341
So-Called Ozomulsion................................... 343
Facial Eczema.......................................    345
SELECTIONS AND ABSTRACTS—Continued:
Abstract of Paper on the Results of One Hundred and Forty-
Seven Operations for Retroversion of the Uterus...  846
Study of the American Medicinal Flora...........    347
The Disguises of Fever..............	..:......... 350
SOCIETY REPORTS:
Atlanta Society of Medicine........ .>.!. j. .....  354
American Medical Association....,<................  357
Reciprocity in Medical Licensure........	..... 361
EDITORIAL..........................’.............. 363,	364
BUSINESS DEPARTMENT.............................       366
BOOK REVIEWS......................................     368
PRESCRIPTIONS....................‘ f1..’. H........... 370
				

